# Salivary C-Reactive Protein: A Non-Invasive Alternative to Serum CRP in Pediatric Acute Appendicitis

**DOI:** 10.3390/molecules30163392

**Published:** 2025-08-15

**Authors:** Klaudio Pjer Milunović, Lada Stanišić, Tomislav Barić, Jakov Meštrović, Davor Todorić, Daniela Šupe Domić, Ana Jerončić, Zenon Pogorelić

**Affiliations:** 1 Department of Pediatric Surgery, University Hospital of Split, 21000 Split, Croatia; 2 Department of Medical Laboratory Diagnostics, University Hospital of Split, 21000 Split, Croatia; 3 Department of Health Studies, University of Split, 21000 Split, Croatia; 4 Department of Research in Biomedicine and Health, School of Medicine, University of Split, 21000 Split, Croatia; 5 Department of Surgery, School of Medicine, University of Split, 21000 Split, Croatia

**Keywords:** salivary biomarkers, c-reactive protein, acute appendicitis, pediatric surgery, non-invasive diagnostics, saliva testing, serum biomarkers, diagnostic accuracy, inflammation markers

## Abstract

Background: The aim of this study was to evaluate the diagnostic potential of salivary C-reactive protein (CRP) as a non-invasive biomarker for acute appendicitis in children and to compare its levels with those found in blood. Methods: Salivary and serum CRP levels were measured in patients with histologically confirmed acute appendicitis (*n* = 46) and a control group with non-specific abdominal pain (*n* = 43). Diagnostic performance was evaluated using receiver-operating characteristic analysis, while the agreement between salivary and serum CRP levels was evaluated using Spearman’s correlation and the Bland–Altman method. Results: Salivary CRP levels were significantly elevated in children with acute appendicitis than in controls (median 35.7 vs. 1.1 mg/L, *p* < 0.001), closely mirroring serum CRP trends (median 44.3 mg/L vs. 1.1 mg/L, *p* < 0.001). Moreover, they demonstrated excellent discriminatory power (Area Under the Curve; AUC = 0.97; 91.3% sensitivity, 95.4% specificity at the optimal cut-off of 6.95 mg/L), comparable to that of serum CRP (AUC = 0.98; 89.1% sensitivity and 95.4% specificity at 10.3 mg/L cut-off). Levels of CRP in serum and saliva were strongly correlated (Spearman’s ρ = 0.963, *p* < 0.001) and overall showed good agreement on Bland–Altman. Although larger discrepancies (>10 mg/L) occurred in 29% of cases, there was no consistent bias favoring either the salivary or serum CRP measurements. Conclusions: Salivary CRP is a promising non-invasive biomarker for diagnosing acute appendicitis in children, demonstrating diagnostic performance closely comparable to that of serum CRP and acceptable agreement between the two measures. This method may reduce the need for invasive blood sampling and streamline early evaluation in pediatric emergency settings.

## 1. Introduction

Acute appendicitis remains the leading cause of emergency surgical intervention in children; however, making an accurate diagnosis continues to pose challenges and can incur significant costs [1,2,3]. It accounts for approximately 20–30% of pediatric cases presenting with acute abdominal pain in surgical emergency settings [4]. Among these cases, the incidence of perforated appendicitis ranges from 12.5% to 45%, and the rate of negative appendectomies still hovers between 5% and 25% [5].

Although numerous diagnostic approaches have been introduced over time, clinicians still largely rely on typical clinical signs and patient history to make a diagnosis [1,6,7]. Thorough history-taking and physical examination remain essential, yet variability in clinical presentation and a wide range of differential diagnoses often delay definitive diagnosis and increase the risk of adverse outcomes [8,9,10]. To improve diagnostic accuracy, supplementary tools such as laboratory tests for inflammatory markers, clinical scoring systems, and imaging techniques are frequently used [1,6,11,12]. Among imaging options, computed tomography (CT) and diagnostic laparoscopy are commonly employed, although they are associated with high costs, longer time requirements, and increased patient discomfort [11].

The majority of routinely available inflammatory biomarkers are currently not sufficiently sensitive or specific to consistently confirm or exclude a diagnosis of acute appendicitis [13,14,15]. The use of a diagnostic screening test, primarily one that is non-invasive, would improve the effectiveness of diagnosis and treatment of a pediatric population with suspected acute appendicitis. C-reactive protein (CRP), routinely measured from blood samples, is one of the most important pro-inflammatory biomarkers in acute appendicitis. CRP functions mainly in innate immune defense. Levels increase in response to inflammation, infection, tissue damage, necrosis, malignancy, and allergic reactions. CRP is a valuable adjunctive inflammatory marker in acute appendicitis, with recent studies reporting a sensitivity of approximately 87% and a specificity of around 77% at a 10 mg/L cut-off in adult patients. Although CRP alone lacks sufficient discriminatory power (Area Under the Curve (AUC) ~0.59), its diagnostic accuracy improves when combined with clinical assessment, scoring systems, and other biomarkers [16]. The majority of CRP originates from the liver, but CRP and IL-6 mRNAs have been detected in gingival tissue samples from periodontitis patients [13].

However, blood collection in children is often challenging due to fear, pain, and limited cooperation. Therefore, there is a need for a less invasive tool that is better or at least equally reliable for use in pediatric clinical practice. As it is a non-invasive, easy-to-perform, and patient-friendly technique, salivary analysis has the potential to provide a novel diagnostic tool for pediatric patients [17,18]. Saliva can be collected via a simple, non-invasive method that does not require specialist training or equipment and minimizes infection risk for the collector compared to blood plasma collection. This makes it more suitable for repeated sampling, collection from difficult patients, and in non-clinical settings [19,20,21,22,23].

Recent studies have identified salivary biomarkers such as leucine-rich α-2-glycoprotein 1 (LRG1) and irisin as potential non-invasive tools for diagnosing acute appendicitis in children. A recent study reported significantly higher salivary LRG1 levels in children with confirmed appendicitis, with a specificity of 100% at a certain cut-off, though sensitivity was limited [24]. Another study found elevated salivary irisin levels in appendicitis patients, with a sensitivity of 90% and specificity of 60%, suggesting promise for clinical use [25]. These findings support further investigation of salivary biomarkers in appendicitis diagnosis.

Although its diagnostic use in acute appendicitis is still under investigation, preliminary studies suggest a positive correlation between salivary and serum CRP concentrations [24,25,26]. In recent reports, salivary CRP analysis represents a feasible non-invasive tool for detecting abnormal serum CRP levels [27]. Small amounts of CRP enter whole saliva from the circulation as a component of the gingival crevicular fluid (GCF) or through salivary glands. Multiple studies reveal associations between salivary CRP and biobehavioral phenomena of interest.

Therefore, this study aims to evaluate the diagnostic potential of salivary CRP as a non-invasive biomarker for acute appendicitis in children and to determine whether its levels are comparable to those of serum CRP, a well-established biomarker, in order to identify a reliable non-invasive alternative or complementary tool.

## 2. Results

### 2.1. Patient’s Clinical Data

The demographic data of the two studied groups are summarized in Table 1. There were no statistically significant differences between the two patient groups regarding age, sex, body weight, and height.

All patients in the acute appendicitis group (*n* = 46) had intraoperative findings consistent with acute appendicitis. Pathohistological analysis confirmed this, showing gangrenous appendicitis in 28 (61%) cases, phlegmonous appendicitis in 13 (28%), and catarrhal appendicitis in 5 (11%), with no cases of innocent appendicitis.

### 2.2. Other Clinical Indicators of Acute Appendicitis

Table 2 summarizes the clinical and laboratory data of the patients. As expected, we observed a significant increase in all inflammatory markers analyzed in the laboratory in patients with acute appendicitis compared to the control group.

### 2.3. CRP from Serum as a Biomarker of Acute Appendicitis

Serum CRP demonstrated strong discriminatory power in identifying children with pathohistologically confirmed acute appendicitis. Median CRP level was significantly higher in the appendicitis group (44.3 mg/L; IQR 21.5–113.1) compared to controls (1.1 mg/L; IQR 0.4–3.9) (*p* < 0.001). The diagnostic performance was excellent, with an AUC of 0.98 (95% CI: 0.92–0.99; *p* < 0.001). The results of the Youden index (J = 0.84; 95% CI: 0.39–0.67) and the corresponding optimal cut-off value (>10.3 mg/L) indicate very good discriminatory performance. At this threshold, the sensitivity and specificity were 89.1% and 95.4%, respectively. Only 2 controls (4.7% out of 43 controls) were falsely classified as having acute appendicitis. The false negative rate was, however, higher, with 5 out of 46 children (10.9%) with confirmed acute appendicitis incorrectly identified as negative.

### 2.4. CRP from Saliva as a Biomarker of Acute Appendicitis

Similarly to the results for serum CRP, CRP levels from saliva also showed strong discriminatory ability. In children with pathohistologically confirmed acute appendicitis, the median salivary CRP level was 35.7 mg/L (IQR 15.9–114.3), significantly higher than in the control group, where the median CRP was 1.1 mg/L (IQR 0.2–2.7) (*p* < 0.001).

**Figure 1:**
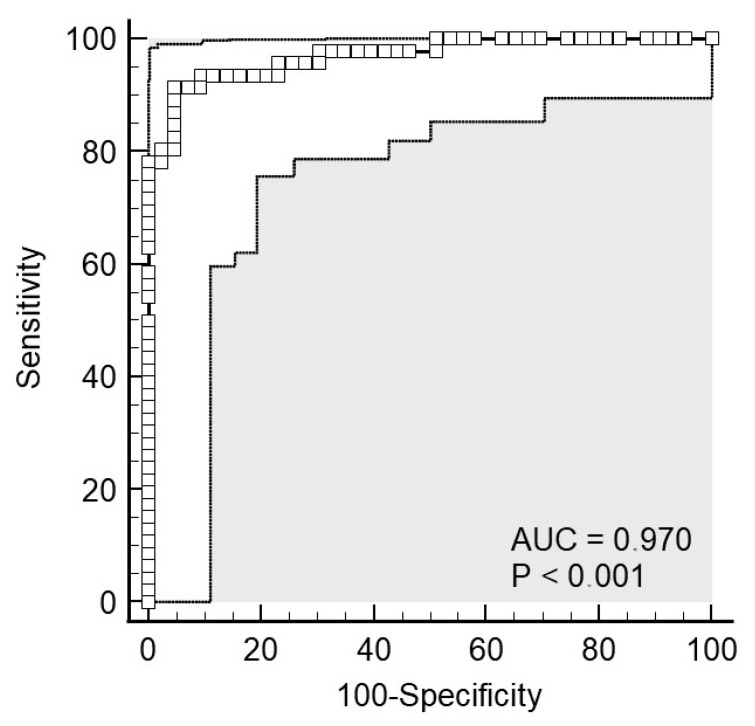
ROC curve for salivary CRP in discriminating acute appendicitis from negative controls presenting with non-specific abdominal pain. The ROC curve is displayed with white rectangles, while the adjacent white areas indicate the 95% confidence intervals around the curve.

Furthermore, salivary CRP levels showed an excellent ability to differentiate children with acute appendicitis from controls (AUC = 0.97; 95% CI 0.91–0.99; *p* < 0.001; Figure 1).

Even after considering uncertainty, the data indicate that the discriminatory ability remains outstanding and similar to that of serum CRP. The Youden index results (J = 0.87; 95% CI: 0.72–0.93) and the corresponding optimal cut-off value (>6.95 mg/L) demonstrate very good discriminative performance. At this cut-off, the sensitivity and specificity were 91.3% and 95.4%, respectively. Only 2 controls (4.7% of 43 controls) were incorrectly classified as having acute appendicitis. The false negative rate was similar to results from CRP serum levels, with 4 of 46 children (8.7%) with confirmed acute appendicitis incorrectly identified as negative.

### 2.5. Agreement Between CRP Levels in Serum and Saliva

**Figure 2:**
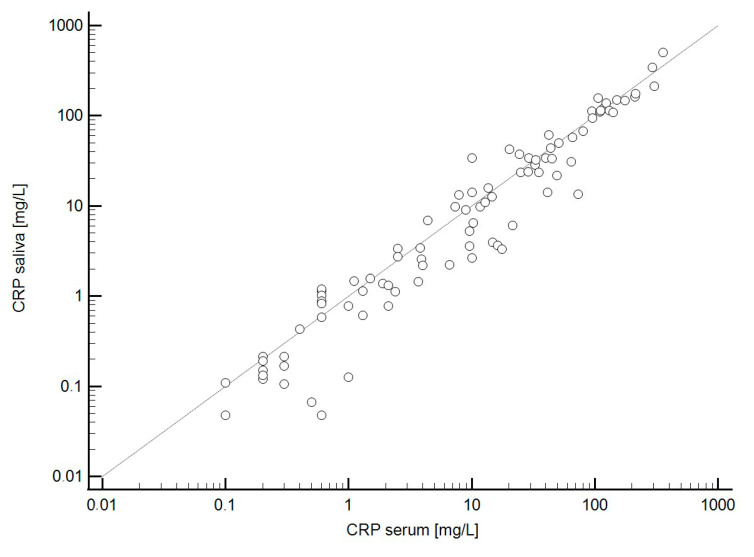
Correlation between CRP concentrations in serum and saliva. Shown is the log–log scatter plot with the identity line.

**Figure 3:**
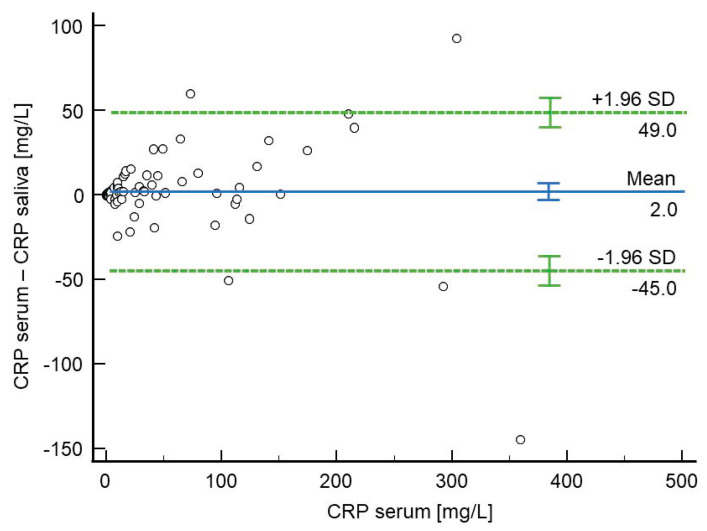
Bland–Altman plot illustrating the agreement between CRP measurements in serum and saliva. The blue capped lines represent the confidence interval around the mean bias (blue horizontal line), while the green capped lines indicate the confidence intervals around the limits of agreement (green horizontal lines).

**Figure 4:**
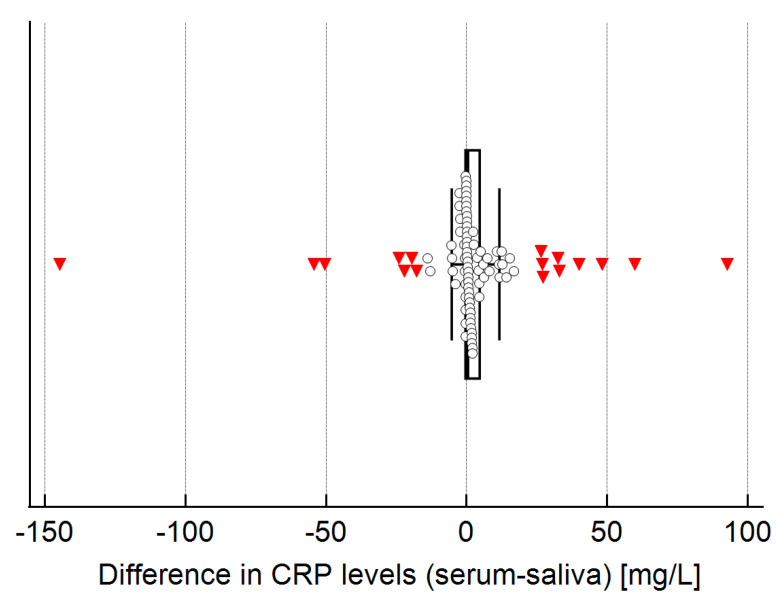
Distribution of differences between serum and saliva CRP levels. Shown is a box plot displaying the distribution of differences in CRP levels between serum and saliva, with individual data points overlaid. Outliers are indicated in red.

**Figure 5:**
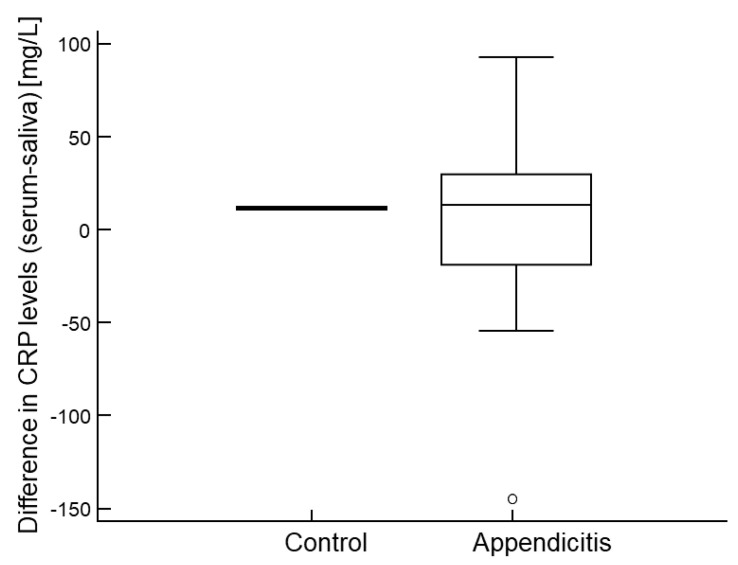
Distribution of large discrepancies (>10 mg/L) in CRP levels between serum and saliva in individual patients, shown by group.

**Figure 6:**
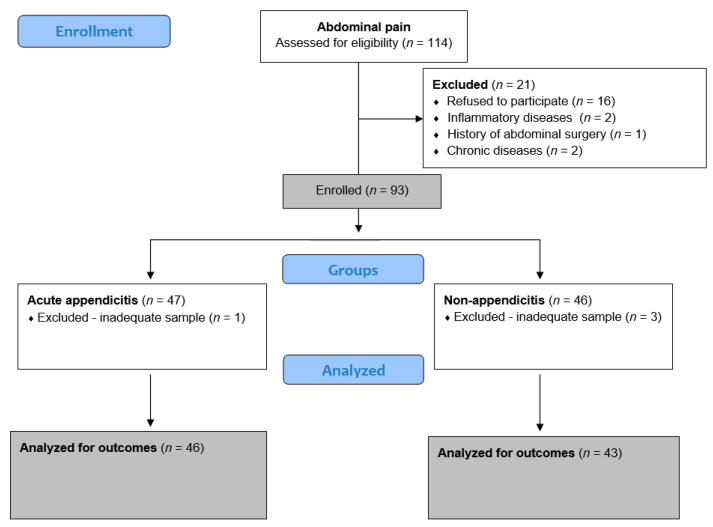
A flow chart of the study.

Serum and salivary CRP levels showed a strong correlation (Spearman’s ρ = 0.963, *p* < 0.001; Figure 2). Bland–Altman analysis demonstrated good average agreement between the two measurements, with a mean difference of only 2 mg/L (95% CI: −3.0 to 7.1). However, the limits of agreement were somewhat broad, ranging from −45.0 to 49.0 mg/L (Figure 3), indicating only moderate overall agreement. Further examination of differences between serum and salivary CRP levels reveals that significant discrepancies (>10 mg/L) sometimes occur (Figure 4). These larger differences—in both directions (positive and negative)—were primarily observed at higher serum CRP levels (Figure 5) and predominantly among children with acute appendicitis. Notably, in 24 out of 26 cases (92%) where the difference exceeded 10 mg/L, the child was diagnosed with appendicitis. The distribution of these large differences, shown in Figure 6, demonstrates that discrepancies occurred in both directions, with no consistent bias that would favor either serum or saliva CRP measurements.

### 2.6. Agreement in Predictive Performance Between Serum and Salivary CRP

Finally, considering the predictive performance using the identified cut-offs, salivary and serum CRP results agreed in 80 of 89 (89.9%) cases. A diagnostic mismatch was observed in nine cases: in three cases, salivary CRP outperformed serum CRP by correctly identifying appendicitis when serum yielded a false negative; in two cases, serum CRP outperformed saliva by correctly identifying appendicitis when saliva yielded a false negative; in two cases, serum CRP produced false positives for appendicitis that saliva did not; and in two cases, saliva CRP produced false positives that serum did not.

## 3. Discussion

The main findings of this study show that saliva collection is a suitable and non-invasive method for use in children and adolescents. Salivary CRP levels were significantly elevated in children with acute appendicitis than in controls, closely mirroring serum CRP trends. Moreover, they demonstrated excellent discriminatory power, comparable to that of serum CRP. Levels of CRP in serum and saliva were strongly correlated and overall showed good agreement on Bland–Altman. Although larger discrepancies (>10 mg/L) occurred in 29% of cases, there was no consistent bias favoring either the salivary or serum CRP measurements.

Various serum biomarkers have been used as diagnostic tools in medicine for a long time [28,29,30]. However, in recent years, saliva has increasingly been employed as a biological sample for analysis related to blood in both children and adults [31,32,33,34,35,36]. Today, saliva diagnostic tests are effectively utilized in infectious diseases, toxicology, forensic medicine, and metabolic conditions [34,35,36]. The benefits of using saliva over serum are numerous, especially in the pediatric population. Collecting saliva is a non-invasive, quick, and simple method. It does not require specialized training for healthcare workers, unlike venipuncture. Saliva collection can be repeated many times, unlike blood sampling, which carries risks of infection and thrombosis. Additionally, saliva does not clot like blood, so anticoagulation is not necessary after collection. This simplifies storage, shipping, and transportation of the samples. Furthermore, it is a painless and stress-free procedure, which is particularly important in children [31,32,33].

Human saliva is an acceptable medium for analysis, as it contains various electrolytes, proteins, immunoglobulins, nucleic acids, and hormones. Recent advances in technology have led to the development of saliva proteomics [37]. Biomarkers traditionally obtained from blood samples have now been identified in saliva, including complement fragments (C3, C4), cytokines (TNF-α, interleukins IL-1, IL-2, IL-6, IL-8), antimicrobial proteins and peptides (S100 protein, lactoferrin, alpha and beta defensins), acute phase proteins (CRP, haptoglobin, transferrin, fibronectin), and immunoglobulins (IgG, IgE, and IgM) [38,39]. Thanks to advancements in genomic technologies, children benefit from these discoveries [37,40,41].

In addition to systemic inflammation, salivary CRP levels may also be influenced by local factors. For instance, oral inflammation, particularly gingivitis and periodontitis, can significantly elevate CRP concentrations via the gingival crevicular fluid, even in the absence of systemic disease. This mechanism may explain some variability in salivary CRP levels, especially in children with mild or early-stage appendicitis [20,27]. Similarly, circadian variation in salivary biomarkers has been reported, including inflammatory markers such as CRP, cortisol, and interleukins. Fluctuations over the day may impact measured values depending on the timing of sample collection. Although our study did not standardize saliva collection time, future research should account for diurnal variation and oral health status to ensure accurate interpretation of salivary CRP data [17,31].

Serum CRP is a sensitive, non-specific biomarker for inflammation and is broadly used in clinical diagnosis of infectious diseases, including appendicitis. Tsai et al. tested several salivary biomarkers, including CRP, IL-6, and IL-10, in children with pneumonia. They used the same ELISA kit (Salimetrics, State College, PA, USA) as in our study. They collected saliva and serum at the initial admission and during a follow-up from pediatric patients with pneumonia and observed that the salivary CRP level was much higher in pediatric patients with pneumonia than in healthy children. Additionally, salivary CRP level was highly correlated with serum CRP levels in pediatric patients with pneumonia. Both serum and salivary CRP levels decreased after treatment [42]. Similar findings were obtained in our study—serum and salivary CRP levels were highly correlated. Tintor et al. compared LRG-1, another non-specific biomarker, in the serum and saliva of children with appendicitis. Their study supported the diagnostic potential of salivary LRG-1 in children with acute appendicitis. The results showed that LRG-1 in saliva enables effective distinction between acute appendicitis and controls (AUC = 0.85; 95% CI 0.76–0.92; *p* < 0.001) [24]. Ramavath et al. investigated serum and salivary CRP as predictors of neonatal sepsis in a cross-sectional analytical study. The AUCs for ROC curve analysis in predicting culture-positive sepsis were 0.72 (95% CI: 0.58 to 0.86, *p* = 0.002) for serum CRP and 0.83 (95% CI: 0.70 to 0.97, *p* = 0.0001) for salivary CRP [43]. The Pearson correlation coefficient between serum CRP and salivary CRP was moderate (*r* = 0.352, 95% CI: 0.135 to 0.537, *p* = 0.002). They found significantly higher salivary CRP levels in neonates with sepsis compared to those without. This was the first study in which CRP in saliva was measured at the bedside using a rapid test, eliminating the need for sample storage and transportation.

Salivary biomarkers in newborns and children are becoming especially useful for initial prognosis and follow-up of various diseases, such as Down syndrome, inflammatory and immune-mediated skin conditions, type 1 diabetes, and familial juvenile systemic lupus erythematosus [44,45,46,47].

Using salivary biomarkers in patients with strongly suspected appendicitis, along with abdominal ultrasound, could reduce unnecessary CT scans that pose radiation risks and potential harm, especially to children. While CRP is a non-specific inflammatory marker, its diagnostic utility in appendicitis lies in its integration with clinical signs, scoring systems, and imaging. In our study, salivary CRP effectively distinguished appendicitis from other causes of abdominal pain, supporting its role as a useful adjunct in the diagnostic pathway.

Several limitations and challenges should be recognized. Firstly, like any other diagnostic tool, salivary biomarkers have limitations in terms of sensitivity and specificity, so the possibility of false positive or false negative results is a concern. For this reason, salivary biomarker data should be interpreted with caution and always considered in conjunction with clinical judgment and standard laboratory findings before making a diagnosis or initiating treatment. Secondly, the diagnostic accuracy of salivary biomarkers can be influenced by the stage and severity of appendicitis as well as by concomitant conditions such as oral infection, inflammation, or recent trauma [42,43]. To ensure consistent and valid results, strict protocols for patient selection, sample collection, and biomarker analysis must be followed. Although the study was adequately powered according to a priori sample size calculations, we acknowledge that the single-center setting and relatively modest sample size taken from underlying population may limit the generalizability of our findings. Therefore, further multi-center studies with larger cohorts are warranted to confirm and extend these results. Another limitation concerns the method of saliva collection. The technique of passive drooling proved to be difficult in children under five years of age, making it difficult to obtain sufficient sample volumes. As a result, this age group was excluded from the current study. In our study, four samples were excluded from the analysis due to insufficient saliva volume. This highlights the importance of collecting a minimum of 1 mL of saliva for reliable analysis, which can be challenging, particularly in younger or dehydrated children. Such limitations should be considered when evaluating the feasibility of salivary diagnostics in pediatric populations. Future research could investigate the use of alternative, age-appropriate collection techniques, such as absorbent swab-based methods, which may facilitate easier sampling and broaden the applicability of salivary CRP testing to children under five years of age.

Additionally, oral health status was not formally assessed in our study. CRP is elevated in the gingival crevicular fluid in the presence of local oral inflammation, such as gingivitis or periodontitis. While all participants were instructed to rinse their mouths before saliva collection, we did not evaluate or exclude individuals based on dental hygiene or the presence of oral infections. As such, we cannot rule out the possibility that undetected gingival inflammation may have confounded some salivary CRP values, particularly in patients with lower systemic inflammatory burden. Nevertheless, given the strong correlation between serum and salivary CRP levels, and the fact that salivary values were not consistently higher than serum values, we do not expect this to have significantly impacted the overall results. Future studies should consider incorporating basic oral health screening to minimize this potential source of bias.

Our findings support the potential integration of salivary CRP testing into clinical pathways for evaluating pediatric abdominal pain. Given its high sensitivity and specificity, salivary CRP could serve as a valuable triage tool in primary care or emergency settings, especially where venipuncture is challenging or access to imaging is limited. Future studies should assess the utility of point-of-care salivary CRP assays in real-time clinical decision-making and explore their cost-effectiveness compared to conventional diagnostic approaches. It is important to emphasize that salivary CRP is not intended to replace clinical judgment or standard diagnostic protocols. Rather, it may serve as a rapid, non-invasive adjunct, particularly useful for triage or early screening, when used alongside clinical scoring systems and imaging findings. The proposed cut-offs represent a starting point for further validation but should not be used in isolation to guide treatment decisions.

## 4. Methods

### 4.1. Study Design and Setting

This prospective, controlled study was conducted at the Department of Pediatric Surgery, University Hospital of Split, Croatia. Between 15 October 2023, and 30 December 2024, pediatric patients older than 5 years who presented to the emergency department with acute abdominal pain and were evaluated for suspected appendicitis were considered for inclusion in the study. Exclusion criteria included the presence of a chronic or inflammatory disease or malignant tumor, a history of invasive abdominal surgery, an age older than 17 years, or a known pregnancy.

### 4.2. Ethical Aspects

The study was conducted in full compliance with the ethical principles outlined in the Declaration of Helsinki, the foundational guideline of the World Medical Association for research involving human participants. Written consent to participate in the study was obtained from all parents or legal guardians of the patients. The study was approved by the Ethics Committee of the University Hospital of Split (approval number: 500-03/23-01/185; date of approval: 20 September 2023). The study has been registered in the *ClinicalTrials.gov* registry under the identifier NCT06051825.

### 4.3. Study Protocol

Data collection included detailed medical histories and demographic information, such as age, sex, weight, and height. Clinical features like symptom duration, right lower quadrant abdominal pain, rebound tenderness, and body temperature were recorded. Intraoperative data included the type of appendicitis based on histopathological findings (catarrhal, phlegmonous, gangrenous, or perforated), surgery duration, complications, and postoperative outcomes, such as hospital stay length and histological confirmation. According to the institution’s standardized protocol for managing suspected acute appendicitis, laboratory tests measured total white blood cell (WBC) count, neutrophil percentage (Neu%), and CRP levels. The Appendicitis Inflammatory Response (AIR) score, a diagnostic tool that combines clinical symptoms, physical signs, and laboratory markers, was used to assess the likelihood of acute appendicitis, incorporating seven predictive variables [48]. All patients also underwent abdominal and pelvic ultrasonography as part of the radiological evaluation. For this case–control study, participants were divided into two groups. The acute appendicitis group (*n* = 46) included patients with a preoperative diagnosis based on clinical assessment, inflammatory markers, and ultrasound findings, confirmed through histopathological analysis after surgery. All group members had laparoscopic appendectomy using a standardized three-port technique, as previously described [49]. The non-appendicitis group (*n* = 43) comprised patients admitted for observation due to non-specific abdominal pain, in whom acute appendicitis was excluded through clinical and diagnostic evaluation. These patients did not need surgical intervention. A schematic overview of the study design is shown in Figure 6.

### 4.4. Blood Collection and Preparation

Venous blood samples were drawn into two types of tubes: one containing a coagulation activator and the other containing the anticoagulant tripotassium ethylenediaminetetraacetic acid (K3 EDTA). Immediately after collection, the samples were transported to the central hospital laboratory. The tube with the coagulation activator was centrifuged to separate the serum, which was then used to measure CRP levels. The sample collected in the K3 EDTA tube was analyzed for total leukocyte and neutrophil counts. These hematological parameters were measured using a standard laboratory hematology analyzer (Advia 2120, Bayer, Leverkusen, Germany). CRP concentrations were assessed using an immunoturbidimetric assay performed on the Cobas C702 chemistry analyzer (Roche, Rotkreuz, Switzerland).

### 4.5. Saliva CRP Collection

Saliva samples were collected from participants in this study at the time of enrolment using the Saliva Passive Drool method with Cryovial and SalivaBios Collection Aid (SCA) (Salimetrics, State College, PA, USA). Prior to saliva collection, patients were instructed to rinse their mouths with water to remove food particles and wait at least 10 min. The ribbed end of the SCA was then placed in a pre-labeled collection vial. Another end of the SCA was placed in the mouth, and up to 1.8 mL of saliva was collected in the vial. It was very important to avoid spitting during saliva collection, as spitting creates bubbles and foam. Once the vial was filled with the required amount, the SCA was removed, and the vial was capped. The vial was then placed in an ice box and processed in the laboratory within 30 min.

### 4.6. Saliva CRP Analysis

After collection, samples were immediately refrigerated and frozen at −80 °C. On the day of assay, samples were thawed, vortexed, and centrifuged for 15 min at 1500 × *g*. Salivary levels of CRP were measured by an enzyme-linked immunosorbent assay (ELISA) kit (Salimetrics, State College, PA, USA). All samples were diluted just before measurement. The amount of diluted sample used per well was 100 µL, and assay ranges were 0−800 pg/mL. To obtain the final CRP sample concentration in pg/mL, we multiply the results by the dilution factor.

### 4.7. Final Diagnosis of the Patients

The diagnosis of acute appendicitis was initially based on intraoperative findings. This was later confirmed by histopathological analysis, which was completed within 2 to 3 weeks after surgery. For patients managed conservatively, acute appendicitis was ruled out after at least 24 h of in-hospital observation, with no surgical intervention deemed necessary during that time. Importantly, the final histopathological diagnosis was blinded to the investigators analyzing salivary CRP levels.

### 4.8. Sample Size Calculation

To determine whether salivary CRP is a promising biomarker for detecting acute appendicitis in children, we calculated the required sample size assuming a large effect size, with an AUC of at least 0.80. The null hypothesis AUC was set at 0.50, with a Type I error of 0.05, a statistical power of 90% and a ratio of positive and negative groups of 1. Based on these parameters, the minimum required sample size was 34.

### 4.9. Statistical Analysis

All statistical analyses were performed using MedCalc statistical software v23.0.6 (MedCalc Software Ltd.; Ostend, Belgium). Qualitative data were summarized using absolute and relative frequencies. Quantitative data were presented as mean ± standard deviation or as median and interquartile range, depending on the distribution of the data. The normality of the data was assessed using the D’Agostino–Pearson test. Depending on the distribution, differences between patient groups for quantitative data were evaluated using the independent *t*-test, Welch’s *t*-test when variances were unequal despite approximate normality, or the non-parametric Mann–Whitney test. For qualitative data, group differences were assessed using the chi-square test. To evaluate the diagnostic performance of CRP from saliva, we conducted receiver-operating characteristic (ROC) curve analysis using the method of DeLong et al. [50], reporting the AUC and the Youden index (J) as measures of test accuracy. Additionally, we calculated the optimal cut-off for group separation while accounting for disease prevalence, assuming, based on the literature, that 7% of children presenting with abdominal pain have acute appendicitis [51,52]. To evaluate the relationship between serum and salivary CRP levels, we first assessed their correlation using Spearman’s rank correlation coefficient. Agreement between serum and salivary measurements was further examined using Bland–Altman analysis, calculating the mean difference with 95% confidence intervals and determining the limits of agreement.

## 5. Conclusions

CRP is an important diagnostic biomarker in acute appendicitis. Although it is non-specific, its sensitivity improves when combined with other indicators. In this study, we demonstrated that salivary CRP not only shows strong diagnostic performance for acute appendicitis but also correlates closely with serum CRP levels, a routinely used biomarker in clinical practice. These findings support its use as a non-invasive alternative or adjunct to serum CRP in pediatric diagnostic pathways.

## Figures and Tables

**Table 1:** Patient characteristics by group.

Variables		Acute Appendicitis (*n* = 46)	Control Group (*n* = 43)	*p*
Age (years), median (IQR)		13 (10–15)	13 (11–14)	0.859 ^†^
Body weight (kg), mean ± SD		162.3 ± 16.3	161.0 ± 13.7	0.603 ^‡^
Body height (kg), mean ± SD		53.3 ± 19.4	51.4 ± 13.5	0.680 ^§^
Sex, *n* (%)	Male	33 (72)	25 (58)	0.181 ^¶^
	Female	13 (28)	18 (42)	

IQR—Interquartile range; SD—Standard deviation. ^†^ Mann–Whitney test, ^‡^ Welch’s *t*-test, ^§^ Independent samples *t*-test, ^¶^ Chi-square test.

**Table 2:** Clinical and laboratory data of the patients.

Variables	Acute Appendicitis (*n* = 46)	Control Group (*n* = 43)	*p* ^†^
Duration of symptoms (h)	28 (24, 48)	36 (24, 48)	0.714
AIR score	9 (7–10)	3 (2, 3)	<0.001
Body temperature (°C)	36.9 (36.8, 37.2)	37.6 (37.2, 38.0)	<0.001
WBC (×10^9^/L)	15.7 (12, 18.6)	7.9 (6.7, 10.4)	<0.001
Neutrophils	79.3 (75.5, 84.7)	56.7 (45.8, 64.5)	<0.001
CRP in serum (mg/L)	44.3 (21.5, 113.1)	1.1 (0.4, 3.9)	<0.001
CRP in saliva (mg/L)	35.7 (15.9, 114.3)	1.1 (0.2, 2.7)	<0.001
Duration of surgery (min)	37.5 (28, 60)	-	
Length of hospital stay (days)	2 (2, 3)	4 (3, 6)	<0.001

AIR—Appendicitis Inflammatory Score; WBC—White blood cells; CRP—C-reactive protein; ^†^ Mann–Whitney U test.

## Data Availability

The data presented in this study are available upon request from the respective author. Due to the protection of personal data, the data are not publicly available.
